# Biocontrol Potential of Essential Oils in Organic Horticulture Systems: From Farm to Fork

**DOI:** 10.3389/fnut.2021.805138

**Published:** 2022-01-13

**Authors:** Yuru Chang, Philip F. Harmon, Danielle D. Treadwell, Daniel Carrillo, Ali Sarkhosh, Jeffrey K. Brecht

**Affiliations:** ^1^Horticultural Sciences Department, University of Florida, Gainesville, FL, United States; ^2^Plant Pathology Department, University of Florida, Gainesville, FL, United States; ^3^Tropical Research and Education Center, University of Florida, Homestead, FL, United States

**Keywords:** bactericidal, disease management, formulation, fungicidal, herbicidal, insecticidal, pest management, phytotoxic

## Abstract

In recent decades, increasing attention has been paid to food safety and organic horticulture. Thus, people are looking for natural products to manage plant diseases, pests, and weeds. Essential oils (EOs) or EO-based products are potentially promising candidates for biocontrol agents due to their safe, bioactive, biodegradable, ecologically, and economically viable properties. Born of necessity or commercial interest to satisfy market demand for natural products, this emerging technology is highly anticipated, but its application has been limited without the benefit of a thorough analysis of the scientific evidence on efficacy, scope, and mechanism of action. This review covers the uses of EOs as broad-spectrum biocontrol agents in both preharvest and postharvest systems. The known functions of EOs in suppressing fungi, bacteria, viruses, pests, and weeds are briefly summarized. Related results and possible modes of action from recent research are listed. The weaknesses of applying EOs are also discussed, such as high volatility and low stability, low water solubility, strong influence on organoleptic properties, and phytotoxic effects. Therefore, EO formulations and methods of incorporation to enhance the strengths and compensate for the shortages are outlined. This review also concludes with research directions needed to better understand and fully evaluate EOs and provides an outlook on the prospects for future applications of EOs in organic horticulture production.

## Introduction

Plant essential oils (EOs) are natural, complex, volatile aromatic, hydrophobic, oily liquids composed of multiple related compounds synthesized in aromatic plants as secondary metabolites (1). Aromatic plants have been appreciated and used for their aromatic and medicinal properties since ancient times. Some aromatic herbs, such as thyme (*Thymus vulgaris*), savory (*Satureja hortensis*), cinnamon (*Cinnamomum verum*), cumin (*Cuminum cyminum*), rosemary (*Salvia rosmarinus*), and clove (*Syzygium aromaticum*) are used worldwide as seasonings to enrich food flavors (2). In addition, many perfumes and fragrances use the fragrant oil of aromatic plants as their main ingredients, for instance, lavender oil, rosemary oil, lemongrass oil, and mint oil, etc. Recently, EOs have also been used as the major therapeutic agents for aroma and massage therapy due to their antiseptic and skin permeability properties. Inhalation, topical application to the skin, massage, and bath are the major methods used in aromatherapy. Aromatherapy utilizes various EOs to treat mental stress and anxiety, as well as numerous other ailments like depression, indigestion, headache and migraine, insomnia, muscular pain, respiratory problems, skin ailments, and swollen joints (2). In a review of the pharmaceutical and therapeutic potential of EOs, Edris (3) summarized published reports of EOs that have shown potential to improve immunity, enhance energy and mental clarity, suppress cancer, and prevent cardiovascular diseases, cholesterol, and diabetes.

Conventional fungicides can cause potential ecotoxicological risks and be hazardous to a wide range of non-target organisms in aquatic systems because they impact basic biological processes that are not unique to fungi (4). The increasing need to control plant pathogens and arthropod pests in organic fruit production promotes the search for safe and effective compounds from natural sources, especially plant-derived compounds. Essential oils have potential in insecticidal, anti-bacterial, anti-fungal, and anti-viral functions as they effectively destroy several pests and pathogens due to the actions of various functional groups such as alcohols, aldehydes, phenolics, terpenes, ketones, and other antimicrobial compounds (1, 5). However, most EOs still need to be handled cautiously and following labeling recommendations given for each situation because EOs may be phytotoxic and cytotoxic at high concentrations (6, 7).

The efficacy of conventional treatments and EO treatments have been compared in some studies. In a study by Zaka et al. (8), four plant essential oils, two plant extracts, two herbicides, and two insecticides were tested against *Tribolium confusum*, a stored grain insect pest. The results suggested that even though the two conventional insecticides, abamectin and cypermethrin, caused higher mortalities in a shorter time, the EOs also showed promising results. Neem EO and citrus plant extract also killed adults of *T. confusum* quicker compared to other treatments. EOs can be better alternatives to highly toxic and hazardous chemicals for stored grain pests management. In another study by Khaliq et al. (9), five EOs (*Calotropis procera, Azadirachta indica, Eucalyptus camaldulensis, Datura stramonium* and *Nicotiana tabacum*) and a conventional fumigant (phosphine gas) were tested at various concentrations individually and synergistically against red flour beetle (*Tribolium castaneum*). The EO of *N. tabacum* (15%) and phosphine gas (500 ppm) caused the highest mortality. The highest synergistic toxic effect were observed for 15% *N. tabacum* and *A. indica* EOs with 500 ppm phosphine gas combinations. The EOs presented promise as alternatives or synergists to improve the efficacy of conventional insecticides. Antibiotics and the EOs may act synergistically by affecting multiple targets, physicochemical interactions, and inhibiting antibacterial-resistance mechanisms. With a better understanding of the mechanisms underpinning synergism, it may be possible to create safe combinations to reduce antibiotic use (10).

**Figure 1:**
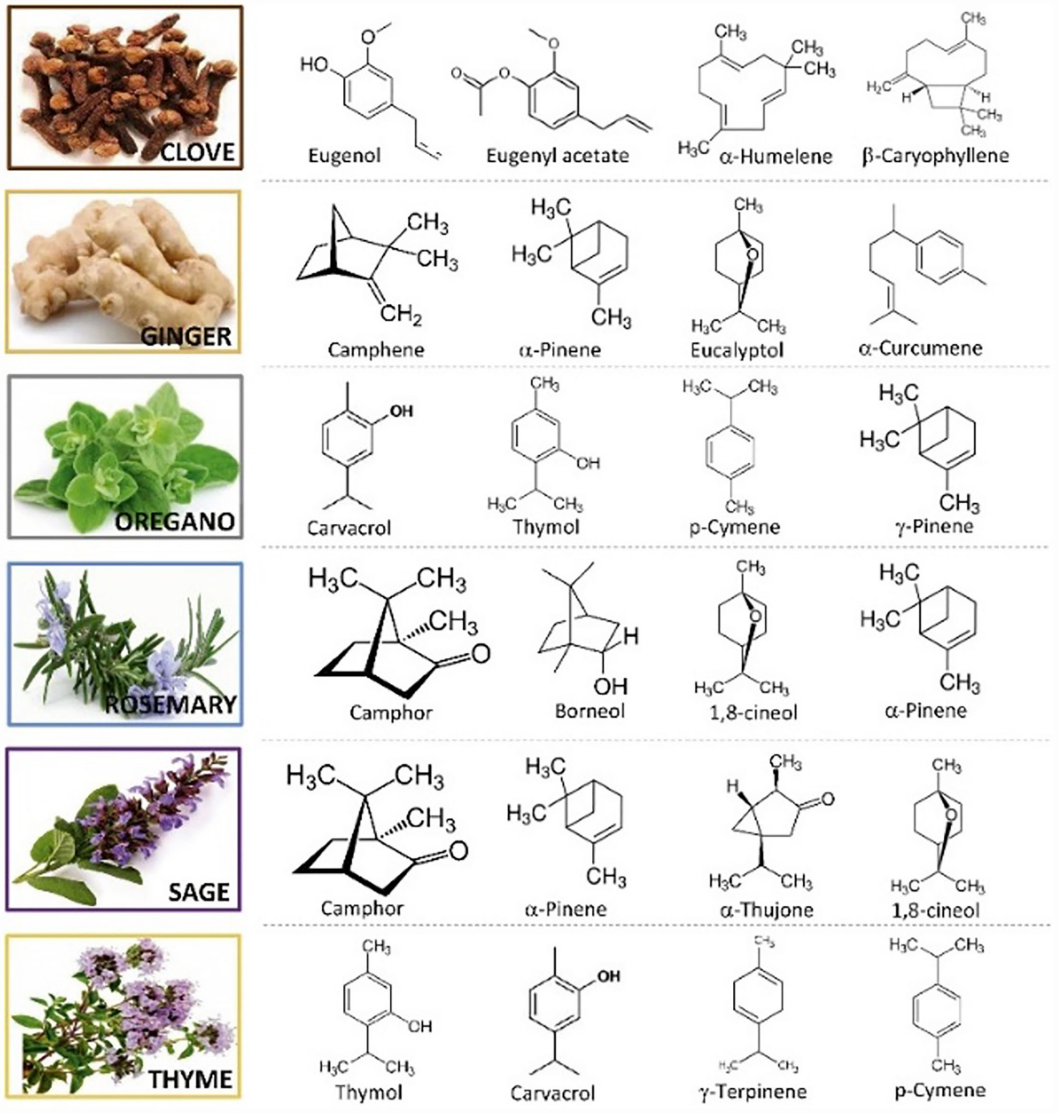
Main antioxidant bioactive compounds found in essential oils (12).

Essential oil composition is mainly determined by genetic, climatic, geographical, and seasonal factors (11). Main antioxidant bioactive compounds of several of the most commonly used EOs are shown in Figure 1. Furthermore, most of the terpenoids and phenols found in EOs have generally fewer toxic effects on plants and mammals than synthetic chemicals (13, 14). The function of EOs in plants is antimicrobial, antioxidant, and insecticidal defense, moreover, their strong flavor makes plants less palatable for herbivores (15, 16).

Various techniques are used for the manufacture and extraction of EOs from plant materials. Traditional methods include cold compression and distillation, along with solvent extraction methods such as maceration, enfleurage, fermentation, percolation, hydro diffusion, and gravity extraction (17, 18). Other promising techniques have been recently applied, such as solvent-free microwave extraction (19), ultrasonic-microwave assisted extraction (20), supercritical fluid extraction (21), subcritical water extraction (22), and ohmic heating-assisted extraction (23). The purpose of these methods is to remove and concentrate the oils from the plant tissue in a pure form. Overall, distillation is the most commonly used method (15), including hydro distillation (24), steam distillation (25), solar distillation (26), and molecular distillation, an extremely low pressure distillation process (27). CO_2_ supercritical fluid extraction is the most efficient method (28). It uses CO_2_ under very high pressure as the solvent; the solvent and extractant separate when the pressure is released.

Many EOs are approved as food additives and fragrance ingredients and are labeled Generally Recognized as Safe (GRAS) in the USA by the Food and Drug Administration (FDA) in CFR - Code of Federal Regulations Title 21 subpart A - General Provisions Sec. 182.20 Essential oils, oleoresins (solvent-free), and natural extractives (https://www.accessdata.fda.gov/scripts/cdrh/cfdocs/cfcfr/CFRSearch.cfm?fr=182.20). Therefore, utilization of EOs as natural preservatives to extend food shelf-life is gaining interest in the food industry (12). Results of studies regarding the use of EOs as antimicrobial agents suggest that EOs can be promising alternatives to synthetic preservatives for postharvest fruits and vegetables (29–31). However, possible negative effects of EOs, such as organoleptic changes (especially smell and taste) and phytotoxicity effects on fresh produce, are sources of concern among handlers and consumers (32).

Horticultural systems of food, fiber, and medicinal crops are complex, multiscale networks inclusive of the entire production, consumption, and post-consumption cycles of thousands of commodities worldwide. Within these systems, the methods of horticultural crop production are influenced by climate, market infrastructure, accessibility to quality farm inputs, regulations, ethical and societal norms, and cultural traditions and values. Organic farming, initially a response to develop alternative approaches to an increasing reliance on synthetic inputs (33) has matured to a global industry whose 2019 market value was estimated at 106.4 billion euros (34). Any farmer may employ the principles of organic farming, which emphasize soil health, animal welfare, and pest prevention, but they may be held accountable to financial penalties if they market their products as organic without the legal validation from a regulatory body. Of the 187 countries reporting adoption of organic practices, 108 have regulatory programs that define and enforce standards of production, processing, and marketing (34).

In the USA, the National Organic Program (NOP) is housed in the Department of Agriculture's (USDA) Agricultural Marketing Service and enforced by USDA accredited agencies (35). The NOP certifies production systems. In part, producers must demonstrate adoption of integrated soil and pest management strategies that conserve natural resources, employ cultural and ecological strategies to manage pests, and use formulated pesticides for pathogens, insects, and weeds only when necessary. Inputs used for crop production are deemed compliant; and raw materials used in inputs must meet the criteria described in the Organic Foods Production Act of 1990 and must be approved by the farmer's certification agency before they are used on the farm to ensure compliance is maintained. In general, synthetic materials are prohibited but there are some exceptions. A current list of all prohibited and approved raw materials allowed for use in organic systems, including those used in food processing, cosmetics, is maintained on the National List (36). Compliant materials and inputs, including formulated pesticides, are readily recognized by farmers if they bear the NOP EPA label or elect to pay a fee for validation of compliance by the Organic Materials Review Institute (OMRI) to use the OMRI-Listed^®^ label.

Compliant pesticides must also satisfy regulatory criteria established by the US Environmental Protection Agency (EPA). Most active and inert ingredients used in compliant pesticides are considered “minimum risk” by the EPA when used according to labeled instructions. Biopesticides are defined by the National Pesticide Information Center as “made of living things, or come from living things, or found in nature” and include three types: microbes, natural substances such as EOs, and plant-incorporated protectants or PIPs, which are genes and proteins introduced to plants via genetic engineering and thus are prohibited by the NOP. Biopesticides made of or from microbes or natural substances have been used in organic farming for decades, but not all commercially-available biopesticides are compliant with the NOP.

The current review aims to provide an overview of the latest studies that have investigated the efficacy of preharvest and postharvest EO applications to manage diseases, arthropod pests, and weeds of horticultural crops. We summarize some of the reasons why EOs are still not in widespread use, list the modes of action of EO products, and outline common EO application methods used today in the food industry. Finally, we highlight gaps in research-based knowledge that should guide the future directions of efforts to increase EO utilization in regulated and non-regulated organic horticulture systems globally.

## Properties of EOs as Broad-spectrum Anti-microbial Agents

### Disease Management

Fungicides are indispensable to global food security as plant diseases are a prime constraint on agricultural and horticultural production (37). Fungicide use on fruits and vegetables accounts for more than 35% of the global pesticide market; while fungicides account for <10% of the total mass of pesticides used in the United States (4). Conventional growers rely on application of synthetic fungicides to mitigate infections and control diseases properly (38). However, abuses of synthetic compounds have resulted in more health hazards and environmental pollution, as well as promoting resistant biotypes of fungal pathogens (39). Currently, a major goal of organic agriculture is to search for alternative disease control methods and concentrate on reaching profitable markets and extending shelf life without compromising product quality and causing environmental pollution (14).

#### Antifungal Properties

Over 19,000 phytopathogenic fungi are known to cause diseases in agricultural and horticultural crops globally (40). Fungal pathogens are the most prevalent among all pathogens and are major causes of quality deterioration of fruits and vegetables. Fungal diseases, which are mainly caused by *Fusarium, Aspergillus*, and *Penicillium* spp., typically cause decay, accelerated ripening, and in some cases an accumulation of mycotoxins (41, 42). Phytopathogenic fungi play a critical role in the profitability, quality, and quantity of fresh products. They are responsible for nearly 30% of all crop diseases and cause quality losses worth billions of dollars worldwide each year (43, 44).

Antifungal properties of EOs against phytopathogenic fungi have been reported in numerous studies. The fungicidal assays of EOs include different methods with either *in vitro* or *in vivo* assessments. The results are mostly expressed as half maximal inhibitory concentration (IC50), minimal inhibitory concentration (MIC), minimum fungicidal concentration (MFC), and zone of inhibition (ZOI) (43). Some commonly studied aspects are mycelial growth inhibition, conidial germination, dry hyphal mass weight, germ tube elongation, and hyphal morphology observation by scanning electron microscopy (SEM) and transmission electron microscopy (TEM).

Essential oils extracted from plants have different effects on various phytopathogenic fungi (Table 1). It was observed on *Alternaria alternata*-inoculated tomato leaves that cinnamon oil and origanum oil vapors reduced necrotic lesions, delayed conidial germination and germ-tube elongation but fennel oil and thyme oil vapors did not. However, these four EOs showed similar antifungal activities against the *in vitro* mycelial growth of *A. alternata* in dose-dependent manners (49). Peppermint oil showed the greatest influence on charcoal rot (*Macrophomina phaseolina*) infection in geranium (*Pelargonium graveolens*), followed by basil oil, while marjoram oil had no effect on the pathogen's growth (111). An *in vivo* postharvest study revealed cardamon (1,000 μL L^−1^) and citronella (750 μL L^−1^) EOs significantly inhibited *Colletotrichum* sp. and *Lasiodiplodia sp*. growth, which cause anthracnose and stem-end rot of papaya, respectively (95). In addition, it was demonstrated that applying each EO or its major constituent resulted in slightly different responses. Thyme oil activity is closely related to its major constituent, thymol. However, the direct application of thymol resulted in a delayed early *A. alternata* infection process, while direct thyme oil only caused a delay in the infection process. Thyme oil from leaves and thymol had MICs of 500 and 250 μg mL^−1^ against *A. alternata*, respectively; while a commercial fungicide Nativo^®^ had a MIC of 1,250 μg mL^−1^ under the same conditions (52). Vapor contact treatment had stronger antifungal activity than direct contact treatment. The EO of *Origanum vulgare*, as well as two of its primary components, thymol and carvacrol, exhibited strong antifungal activity against *Botrytis cinerea*. From an *in vitro* study, using direct contact assays, thymol (17.56 mg L^−1^) had the lowest EC50 against *B. cinerea* mycelial growth, followed by carvacrol (26.22 mg L^−1^) and the EO (52.92 mg L^−1^). In vapor contact assays, the EC50 values were substantially lower than those in direct contact assays, with thymol (0.94 mg L^−1^) having the lowest EC50 value, followed by carvacrol (1.61 mg L^−1^), and the EO (16.44 mg L^−1^). In vapor contact assays, the EO exhibited an MIC of 31.25 mg L^−1^ against *B. cinerea* spore germination and an MFC of 62.5 mg L^−1^. Thymol and carvacrol had identical MIC and MFC values of 7.81 and 15.63 mg L^−1^, respectively. From an *in vivo* study, compared to the control, EO vapor contact treatment reduced the deterioration of cherry tomatoes by 70.44% at 62.5 mg L^−1^. Fruit decay was reduced by 96.39% at a concentration of 250 mg L^−1^. Thymol and carvacrol at 62.5 mg L^−1^ suppressed the development of *B. cinerea* lesions by 94.13 and 95.37%, respectively, whereas they both completely suppressed the development of *B. cinerea* at 125 mg L^−1^ (85).

**Table 1:** Examples of EOs acting against phytopathogenic fungi/oomycetes.

**Essential oil distilled from plant species**	**Target fungi**	**Disease caused**	**References**
*Cymbopogon nardus*	*Alternaria alterna*	Black rot and leaf bligt	(45)^ab^
*Thymbra spicata, Lavandula stoechas, Foeniculum vulgare*			(46)^a^
*Laurus nobilis*			(47)^ab^
*Mentha x piperita*			(48)^a^
Cinnamon oil, origanum oil			(49)^ab^
*Cinnamomum zeylanicum, Eugenia caryophyllus*			(50)^ab^
*Mentha arvensis*			(51)^ab^
*Thymus vulgaris*			(52)^ab^
*Juniperus polycarpos*			(53)^a^
*Armeniaca sibirica*	*Alternaria brassicae*		(54)^ab^
*Satureja hortensis*	*Alternaria citri*		(55)^a^
*Artemisia* spp.	*Alternaria solani*		(56)^a^
*Lippia alba*			(57)^a^
*Simmondsia chinensis, Zingiber officinale Roscoe, Allium sativum, Syzygium aromaticum, Sesamum indicum, Eucalyptus glabulus, Cinnamon zylanicum, Ricinus communis, Citrus limon, Brassica nigra*			(58)^ab^
*Armeniaca sibirica*			(54)^ab^
*Allium sativum*	*Alternaria tenuissima*		(59)^a^
*Daucus carota*	*Alternaria triticina*		(60)^a^
*Curcuma longa*	*Aspergillus flavus*	Ear rot	(61)^ab^
*Thymus vulgaris*			(62)^a^
*Rosmarinus officinalis*			(63)^a^
*Perilla frutescens*			(64)^a^
*Ammodaucus leucotrichus*			(65)^a^
*Zhumeria majdae*			(66)^a^
*Perilla frutescens*	*Aspergillus glaucus*	Black mold	(67)^a^
Thyme oil, clove oil	*Aspergillus niger*		(68)^ab^
*Rosmarinus officinalis*			(69)^a^
*Eugenia caryophyllata, Piper nigrum*			(70)^ab^
*Ammodaucus leucotrichus*	*Aspergillus ochraceus*		(65)^a^
*Cymbopogon citratus, Eucalyptus globulus, Origanum vulgare, Ruta graveolens, Salvia officinalis, Satureja Montana*	*Aspergillus parasiticus*		(71)^a^
*Allium cepa*	*Aspergillus* spp.		(72)^a^
*Cymbopogon citratus, Thymus vulgaris, Origanum vulgare, Syzygium aromaticum*			(73)^ab^
*Zhumeria majdae*	*Aspergillus tubingensis*	Leaf spot	(66)^a^
*Cinnamomum tamala*	*Bipolaris australiensis*		(74)^a^
*Lippia sidoides*	*Bipolaris maydis*		(75)^ab^
*Heteranthera reniformis*	*Bipolaris oryzae*		(76)^a^
*Rosmarinus officinalis*			(77)^a^
*Daucus carota*	*Bipolaris sorokiniana*		(60)^a^
*Ocimum basilicum*	*Bipolaris* spp.		(78)^a^
*Myrtaceae* spp.	*Biscogniauxia mediterranea*	Charcoal canker	(79)^a^
*Mentha spicata, Cymbopogon martini*	*Botrytis cinerea*	Gray mold	(80)^ab^
*Syzygium aromaticum, Thymus vulgaris*			(81)^a^
*Melaleuca alternifolia*			(82)^ab^
*Tetraclinis articulata*			(83)^ab^
*Mentha* sp.			(84)^ab^
*Origanum vulgare*			(85)^ab^
*Litsea cubeba*			(86)^a^
*Satureja montana, Thymus vulgaris*			(87)^b^
*Zhumeria majdae*			(66)^a^
*Cinnamon zeylanicum, Zataria multiflora*			(88)^ab^
*Cinnamomum tamala*	*Choanephora cucurbitarum*	Flower and fruit rot	(74)^a^
*Zhumeria majdae*	*Cladosporium cladosporioides*	Leaf spot	(66)^a^
*Thymus vulgaris, Cinnamomum zeylanicum*	*Colletotrichum acutatum*	Anthracnoe	(89)^ab^
*Cinnamomum* sp.			(90)^a^
*Thymus vulgaris, Salvia officinalis, Mentha piperita*			(91)^ab^
*Schinus molle*	*Colletotrichum gloeosporioides*		(92)^a^
*Bunium persicum*	*Colletotrichum lindemuthianum*		(93)^ab^
*Thymus vulgaris*	*Colletotrichum musae*		(94)^ab^
*Elettaria cardamomum, Cymbopogon nardus*	*Colletotrichum* sp.		(95)^a^
*Juniperus polycarpos*	*Colletotrichum trichellum*		(53)^a^
*Juniperus polycarpos*	*Curvularia fallax*	Black sheath spot	(53)^a^
*Cedrus deodara*	*Curvularia lunata*	Leaf spot	(96)^a^
*Cymbopogon citratus*			(97)^ab^
*Juniperus polycarpos*	*Cytospora sacchari*	Cytospora canker	(53)^a^
*Piper auritum*	*Fusarium equiseti*	Root rot	(98)^a^
*Zingiber officinale*	*Fusarium graminearum*	Fusarium head blight	(99)^a^
*Baccharis dracunculifolia, Pogostemon cablin*			(100)^a^
*Juniperus polycarpos*	*Fusarium oxysporum*	Fusarium wilt	(53)^a^
*Syzygium aromaticum*			(101)^ab^
*Eugenia caryophyllata, Piper nigrum*			(70)^ab^
*Piper auritum*			(98)^a^
*Cymbopogon* sp.	*Fusarium solani*	Root rot	(102)^ab^
*Mentha piperita*	*Fusarium sporotrichioides*	Fusarium head blight	(103)^a^
*Allium cepa*	*Fusarium* spp.		(72)^a^
*Eucalyptus camaldulensis*			(104)^a^
*Zhumeria majdae*			(66)^a^
*Rosmarinus officinalis*	*Fusarium verticillioides*	Stalk rot and ear rot	(105)^a^
*Curcuma longa*			(106)^a^
*Mentha × piperita*	*Geotrichum citri*	Sour rot	(107)^a^
*Elettaria cardamomum, Cymbopogon nardus*	*Lasiodiplodia* sp.	Dieback and blights	(95)^a^
*Melaleuca alternifolia*	*Lasiodiplodia theobromae*		(108)^ab^
*Juniperus polycarpos*	*Macrophomina phaseolina*	Charcoal rot	(53)^a^
*Mentha* sp., *Lippia gracilis*			(109)^a^
*Mentha viridis, Mentha piperita*			(110)^ab^
*Mentha piperita, Ocimum basilicum*			(111)^ab^
*Lavandula* spp.	*Monilinia fructicola*	Brown rot	(112)^ab^
Tea tree oil			(113)^ab^
*Origanum vulgare*	*Monilinia laxa*		(114)^b^
*Syzygium aromaticum*	*Penicillium digitatum*	Green mod	(115)^ab^
			(116)^ab^
*Citrus aurantium*			(117)^ab^
*Cymbopogon citratus*	*Penicillium expansum*		(118)^b^
*Allium sativum*	*Penicillium funiculosum*		(119)^a^
*Melaleuca alternifolia*	*Penicillium griseofulvum*		(120)^a^
*Citrus aurantium*	*Penicillium italicum*		(117)^ab^
*Thymus vulgaris*	*Penicillium paneum*		(121)^ab^
*Allium cepa*	*Penicillium* spp.		(72)^a^
*Melaleuca alternifolia*	*Penicillium verrucosum*		(120)^a^
*Cinnamomum zeylanicum*	*Phytophthora colocasiae*	Leaf blight	(122)^a^
*Allium sativum*	*Phytophthora nicotianae*	Root and fruit rot, leaf and stem infection	(123)^ab^
*Thymus vulgaris, Satureja hortensis*	*Raffaelea quercus-mongolicae*	Oak wilt	(124)^a^
*Cinnamomum tamala*	*Rhizoctonia solani*	Leaf spot and root rot	(74)^a^
*Lippia alba*			(125)^ab^
*Thymus vulgaris, Satureja hortensis*			(124)^a^
*Lippia sidoides*	*Rhizopus stolonifer*	Soft rot	(126)^ab^
*Citrus sinensis*			(127)^a^
*Cinnamon zeylanicum, Zataria multiflora*			(88)^ab^
*Zhumeria majdae*	*Sclerotinia sclerotiorum*	Soft rot	(66)^a^
*Ziziphora clinopodioides*			(128)^ab^
*Piper aduncum*			(129)^a^
*Murraya paniculata*			(130)^a^
*Origanum dubium*			(131)^a^
*Daucus carota*	*Ustilago segetum*	Loose smut	(60)^a^
*Thymus* sp.	*Verticillium dahlia*	Verticillium wilt	(132)^ab^
*Cinnamomum* spp.	*Villosiclava virens*	Rice false smut	(133)^a^

*Only treatments that displayed staitically significant antifungal effects are included; consult the references for treatment details*;

a *in vitro study*;

b *in vivo study*;

ab *both in vitro and in vivo study*.

The mechanisms of EO antifungal activity could be: (1) Cell wall and membrane disruption leading to cell membrane permeability change and leakage of cell cytoplasm. The ultrastructure analysis demonstrated that thymol, major constituent of thyme oil, acted at the cellular level against fungi by disrupting cell wall and plasma membrane with subsequent cytoplasm disorder (52). The results from SEM and TEM revealed that EO-damaged hyphae cell membranes and changed the cell membrane permeability, leading to the changes in the cytoplasm components, such as reducing soluble sugars, proteins, and ergosterol (86). Another study using SEM and TEM confirmed that mint oil could disrupt cell walls and destroy the ultrastructure of hyphae and conidia, resulting in cellular nucleic acids and proteins leakage and marked shriveling and crinkling of the hyphae and conidia (84). (2) Influence cell energy metabolism pathway. In one *in vitro* and *in vivo* study on EOs against *Aspergillus niger* it was further reported that EOs could probably inhibit glycolysis, which in turn influenced cell energy metabolism of fungal pathogens (68). In another study, it was shown that EOs can disrupt the integrity of plasma membranes and cause mitochondrial dysfunction, inducing metabolic stagnation in fungi. Moreover, EOs can modulate mycotoxin gene expression in the aflatoxin biosynthesis pathway on *Aspergillus flavus* (61). (3) Defense dysfunction. Essential oils could destroy the normal morphology and activities of cell wall and membrane and cause defense dysfunction against stress response. Analyses of multiple metabolic pathways illustrated that spore development, membrane permeability, oxidative stress, and amino acid metabolism were all disturbed (64). (4) Accumulation of ROS. It was reported that EOs could stimulate accumulation of ROS in mycelia and spores and cause a rapid increase in intracellular reactive oxygen species levels (113). In other research, it was shown that intracellular ROS generated by EOs damaged cell membranes and this might have caused pathogen cell death (124). (5) Anti-aflatoxigenic effect. Thyme EO significantly reduced the aflatoxin B1 (AFB1) production of *Aspergillus flavus in vitro*. This anti-aflatoxigenic property was attributed to the down-regulation of the secondary metabolism gene *laeA* and to the modulation of hydrolase gene expression involved in fungal colonization and establishment (62). Rosemary EO could reduce the production of ergosterol and the biomass of mycelium, and inhibit the production of aflatoxins B1 and B2, indicating that the antiaflatoxigenic effect of rosemary EO is independent of its antifungal effect and is likely due to its direct action upon toxin biosynthesis (63). (6) Regulate specific gene expression in the host. The expression of the pathogenesis-related (PR) gene PR-8 in apple was induced by 2.5-fold by EOs compared to untreated inoculated fruit, which suggested that EOs induced resistance against pathogens through the priming of defense responses (87).

#### Antibacterial Properties

Bacteria causing diseases on plants also have a considerable economic impact. Plant pathogenic bacteria survive in diverse environments, both in plants, as pathogens, and outside their hosts as saprophytes. About 350 bacteria, which are pathovars or subspecies belonging to the phyla Proteobacteria, Actinobacteria, and Firmicutes, are known to be phytopathogenic [(158); Table 2].

**Table 2:** Examples of EO acting against phytopathogenic bacteria.

**Essential oil distilled from plant species:**		**Target bacteria**	**Disease caused:**	**References**
*Satureja hortensis*	Gram-positive	*Clavibacter michiganensis*	Bacterial wilt and canker	(134)^ab^
*Origanum vulgare*				(135)^ab^
*Allium sativum*				
*Ocimum basilicum*				
*Cinnamomum zeylanicum*				
*Syzygium aromaticum*				
*Thymus vulgaris*				
*Allium sativum*		*Rhodococcus fascians*	Leafy gall syndrome	(136)^a^
*Ocimum ciliatum*				(137)^a^
*Teucrium polium*		*Streptomyces scabies*	Common scab	(138)^a^
*Allium sativum*	Gram-negative	*Agrobacterium tumefaciens*	Crown gall	(136)^a^
*Eriocephalus africanus*				(139)^a^
*Cinnamomum verum*				(140)^a^
*Pinus halepensis*				(141)^a^
*Dysphania ambrosioides*				(142)^a^
*Ocimum ciliatum*		*Agrobacterium vitis*	Crown gall	(137)^a^
*Ocimum ciliatum*		*Brenneria nigrifluens*	Canker	(137)^a^
*Teucrium polium*				(138)^a^
*Eriocephalus africanus*		*Dickeya solani*	Soft rot	(139)^a^
*Pinus halepensis*				(141)^a^
*Citharexylum spinosum*				(143)^a^
*Bougainvilla spectabilis*				
*Allium sativum*		*Erwinia amylovora*	Fire blight	(136)^a^
*Apium graveolens*				(144)^ab^
*Curcuma longa*				
*Eriocephalus africanus*				(139)^a^
*Dysphania ambrosioides*				(142)^a^
*Eugenia caryophylata*		*Erwinia carotovora*	Black stem and soft rot	(145)^ab^
*Cinnamomum zelanicum*				
*Datura metel*				
*Origanum vulgare*		*Erwinia rhapontici*	Crown rot	(146)^a^
*Cinnamomum cassia*		*Klebsiella pneumoniae*	Block root respiration	(147)^a^
*Teucrium polium*		*Pantoea agglomerans*	Bacterial blight	(138)^a^
*Ocimum ciliatum*		*Pantoea stewartii*	Stewart's wilt and leaf blight	(137)^a^
*Pinus halepensis*		*Pectobacterium atrosepticum*	Soft rot	(141)^a^
*Bougainvilla spectabilis*		*Pectobacterium carotovorum*	Soft rot	(143)^a^
*Eucalyptus globulus*		*Pseudomonas aeruginosa*	Bacterial root rot	(148)^a^
*Thymus vulgaris*				(149)^a^
*Origanum majorana*				
*Eriocephalus africanus*		*Pseudomonas cichorii*	Bacterial leaf blight	(139)^a^
*Thymus serpyllum*		*Pseudomonas savastanoi*	Bacterial canker	(150)^a^
*Origanum syriacum*				
*Satureja hortensis*		*Pseudomonas syringae*		(134)^ab^
*Ocimum ciliatum*				(137)^a^
*Cynara cardunculus*				(151)^a^
*Dysphania ambrosioides*				(142)^a^
*Lantana camara*		*Ralstonia solanacearum*	Bacterial wilt	(152)^a^
*Corymbia citriodora*				(153)^a^
*Cinnamomum* spp.				(154)^a^
*Tagetes patula*				(155)^a^
*Solanum torvum*				(155)^a^
*Teucrium polium*				(138)^a^
*Pinus halepensis*				(141)^a^
*Ocimum ciliatum*				(137)^a^
*Origanum vulgare*				(135)^ab^
*Allium sativum*				
*Ocimum basilicum*				
*Cinnamomum zeylanicum*				
*Syzygium aromaticum*				
*Thymus vulgaris*				
*Thymbra spicata*		*Rhizobium radiobacter*	Crown gall	(150)^a^
*Thymus serpyllum*				
*Origanum syriacum*				
*Teucrium polium*				(138)^a^
*Teucrium polium*		*Rhizobium vitis*	Crown gall	(138)^a^
*Cinnamomum cassia*		*Serratia marcescens*	Leaf Spot	(147)^ab^
*Thymus vulgaris*		*Solanum lycopersicum*	Late blight	(156)^ab^
*Cymbopogon citratus*				
*Satureja hortensis*		*Xanthomonas axanopodis*	bacterial canker	(134)^ab^
*Thymus vulgaris*				(156)^ab^
*Cymbopogon citratus*				
*solanum torvum*				(155)^a^
*Satureja hortensis*		*Xanthomonas campestris*	Black rot	(134)^ab^
*Teucrium polium*				(138)^a^
*Origanum vulgare*				(146)^a^
*Ocimum ciliatum*		*Xanthomonas citri*	Citrus bacterial canker	(137)^a^
*Citrus aurantium*				(157)^a^
*Citrus aurantifolia*				
*Ocimum ciliatum*		*Xanthomonas oryzae*	Bacterial blight	(137)^a^
*Cynara cardunculus*		*Xanthomonas perforans*	Bacterial spot	(151)^a^

*Only treatments that displayed statistically significant antibacterial effects are included; consult the references for treatment details*;

a *in vitro study*;

*^b^in vivo study*;

ab *both in vitro and in vivo study*.

The antibacterial activities of EO are mostly expressed in MIC, minimal bactericidal concentration (MBC), and ZOI. Regarding *in vitro* antibacterial activities tests, overall Gram-positive organisms are more susceptible to EO compared with Gram-negative organisms, which is due to the structure of the cellular membrane. It was reported that the susceptibility of Gram-positive bacteria was observed to be greater than that of Gram-negative bacteria when treated with EO distilled from *Citrus medica* (159), *Ocimum basilicum* (160), *Mentha spicata* (161), *Nepeta ucrainica* (162), *Zanthoxylum schinifolium* (163), and *Zingiber officinale* (164).

The antibacterial activity of basil (*Ocimum ciliatum*) EO, as a new source of methyl chavicol, was tested against ten important phytopathogenic bacteria. The antibacterial test results indicated that the EO had antibacterial activity against all of the bacteria tested. The most vulnerable bacterium was *Brenneria nigrifluens*, while the most resistant bacterium was *Pseudomonas tolaasii*, based on the ZOI values. Moreover, the EO had the lowest MIC values against *Ralstonia solanacearum* and the lowest MBC was found to have the strongest bactericidal property against the *Xanthomonas citri* (137). Eleven EOs were screened for antibacterial activities and abilities to influence the growth and virulence factors of *Erwinia amylovora*, causing fire blight. According to ZOI values, *Foeniculum vulgare* and *Pimpinella anisum* EOs showed strong antibacterial activity and *Artemisia aucheri* and *Heracleum persicum* EOs had moderate antibacterial activity. The other seven EOs did not show substantial growth inhibition but could reduce the production of virulence factors in *E. amylovora* at non-lethal concentrations. Both *A. aucheri* and *F. vulgare* EOs indicated the highest bacteriostatic (MBC 15.6 μg/mL) and bactericidal (MIC 7.8 μg/mL) activities. In contrast, *Citrus sinensis* and *Citrus aurantifolia* exhibited minimal bacteriostatic (MBC 250 μg/mL) and bactericidal (MIC 250 μg/ mL) activities. From an *in vivo* study, EOs of *Apium graveolens* (celery seed) and *Curcuma longa* (turmeric) demonstrated the greatest reduction in the impact of *E. amylovora* virulence factors. They reduced disease progression of *E. amylovora* on immature pear fruit by 41.71 and 30.17%, and disease progression in pear seedling shoots by 26.9 and 16.7%, respectively (144).

The mechanisms of EO antibacterial activities have been widely studied. In a recent study, the mechanisms of antibacterial activity of finger citron essential oil were investigated by observing changes of bacteria morphology according to scanning electron microscopy, time-kill analysis, and permeability of cell and membrane integrity. Morphology of *Escherichia coli* and *Staphylococcus aureus* were changed and damaged more seriously with higher concentration and longer exposure time to finger citron EO. It significantly suppressed the growth rate of surviving bacteria and led to lysis of the cell wall, intracellular ingredient leakage, including small ions, nucleic acids, and proteins, and finally cell death (159). Cinnamon EO was reported to cause the leakage of small electrolytes, rapidly increasing the electric conductivity of *S. aureus* and *E. coli* within the first few hours of exposure, decreasing the bacterial metabolic activity 3–5-fold. Furthermore, the concentration of proteins and nucleic acids in cell suspension also increased with increased cinnamon EO (165). Under the transmission electron microscope, *Cinnamomum longepaniculatum* leaf EO decreased cell size, and ruptured the cell walls and cell membranes of treated bacteria. Moreover, nucleoplasm was reduced and gathered onto the side, which might be attributed to its hydrophobicity (166). In addition, as a result of post-contact effects, cell constituents release assays, and ultrastructural analysis revealed that the loss of integrity of the cell membranes and vital intracellular constituents could be one of the mechanisms of action of the green huajiao (*Zanthoxylum schinifolium*) EO against selected foodborne pathogens (163). Electron microscopy observation indicated that fennel seeds EO disrupt membrane integrity, according to the leakage of electrolytes and the losses of protein and sugar contents of targeted bacteria (163). In other research, it was shown that the nanoemulsion amplified the antibacterial activity of *Thymus daenensis* EO against *E. coli* by increasing the EO ability to disrupt cell membrane integrity. The results were investigated by measuring potassium, protein, and nucleic acid leakage from the cells, and by electron microscopy (167). *Litsea cubeba* EO can inhibit the respiratory metabolism, the hexose monophosphate pathway and its key enzyme (glucose-6-phosphate dehydrogenase) of methicillin-resistant *Staphylococcus aureus*. Moreover, citral, the main component of *Litsea cubeba* EO, could further form chimeras with DNA of methicillin-resistant *Staphylococcus aureus* to inhibit its biological activity (168).

In summary, EO antibacterial mechanisms might include loss of integrity of the cell walls and membranes, leakage of electrolytes, loss of intracellular constituents, increase of bacteria electrical conductivity and nucleic acid concentration in cell suspension, and inhibition of respiratory metabolism decrease bacterial metabolic activity.

### Arthropod Pest Management

Indiscriminate use of conventional synthetic chemical insecticides has caused different types of environmental and toxicological problems, such as environmental pollution, toxicity to non-targeted organisms, and development of pesticide resistance. Therefore, it is important to identify more biodegradable alternatives that are less persistent in the environment than chemical insecticides (169). Interest in the potential use of natural and botanical products for pest management, such as EOs or their derivatives, has grown dramatically in the past two decades due to their lower mammalian toxicity and faster environmental degradation (170–172).

EOs have antimicrobial or insecticidal properties that can protect plants from herbivores and microorganisms. In line with the known synergistic effects of complex EO mixtures, the knockdown and mortality rates, and biocidal activity against some adult dipteran insects increased by up to 100% for certain EO mixtures compared to individual EOs (173). Another study indicated that citral can be synergistic to limonene and geranyl acetate when their concentrations increase in the mixture. Moreover, the binary mixture of the two major constituents of lemongrass oil, citral and limonene, displayed synergistic cytotoxicity on an ovarian cell line of the cabbage looper (174). However, another comparison of toxicity and deterrent activity with artificial blends as binary mixtures revealed that synergism was not a generalized phenomenon and both species and blend specific variations can occur (175).

Essential oils are believed to interfere with basic metabolic, biochemical, physiological and behavioral functions of insects. However, little is known about their complete modes of action (169, 176). The mono- and sesquiterpenoid constituents of EOs are fast-acting neurotoxins in insects and related arthropods, possibly interacting with multiple types of receptors in their nervous system (171). These compounds also display potentially important sublethal behavioral effects on arthropods, such as repellence; oviposition deterrence; ovicidal, larvicidal pupicidal and antifeedant effects (169). Different EOs may work via different mechanisms. The acute toxicity and feeding deterrent activity of ten common EOs against three postharvest stored-grain pests (*Sitophilus oryzae, Tribolium castaneum*, and *Rhyzopertha dominica*) were evaluated (175). Thymol, carvacrol, eugenol, and trans-anethole showed differential species-specific toxicity, and acted as acute toxins rather than feeding deterrents. However, linalool was a general feeding deterrent against all three species. The antifeedant activity could be due to physiological toxicity rather than interaction with gustatory receptors. The authors further reported combined toxic and antifeedant effects for various combinations of anethole, carvacrol, or linalool. Moreover, the observed decreased beetle mortality, but increased feeding deterrence, implied that the physiological toxicity induced by acute toxins synergized with the deterrent activity of a compound in a mixture, but the dose may not be sufficient to kill (175).

Essential oils have also been used as insect attractants in pest control programs. *Coriandrum sativum* and *Nerium indicum* EOs were strong attractants of both adults and nymphs of *Cyrtorhinus lividipennis*, a major predator of the rice planthopper (177); Thus, these OEs can be used in augmentative biological control against rice pests. In addition, EOs are used as lures for detecting and monitoring invasive ambrosia beetles (178, 179).

Essential oils were shown to control preharvest and postharvest phytophagous insects during their development, growth, and adult emergence (169). As of this writing, screening, discovery, and demonstration of bioactivity have been reported in numerous studies of various EOs against various insect pests (180). Ebadollahi et al. (181) summarized in their review paper that the main components in the EOs extracted from the *Lamiaceae* plant family exhibit insecticidal effects. For instance, terpinen-4-ol displayed contact and fumigant toxicity against adults of *Cimex lectularius* (182), α-pinene showed fumigant and contact toxicities and repellency against adults of *T. castaneum* (183), terpinolene demonstrated larvicidal and pupicidal activity against *Culex quinquefasciatus* (184), and α-terpineol exhibited fumigant toxicity against the adults of *Sitophilus granaries* (185). In addition, Stepanycheva et al. (186) reported that the EOs obtained from *Mentha pulegium* and *Thymus mastichina* showed acute toxicity effects against western flower thrips (*Frankliniella occidentalis* Perg.) by fumigation.

EOs have also been used against pestiferous mites and nematodes. Lemongrass (*Cymbopogon citratus*) oil showed promising miticidal activity and ovicidal effects against *Sarcoptes scabiei* (187). Besides, Ozdemir and Gozel (188) tested 10 EOs and discovered *Lavandula officinalis, Artemisia absinthium, Piper nigrum, Citrus bergamia* and *Mentha arvensis* have the most nematicidal effects against the root-knot nematode *Meloidogyne incognita*. Other than the commonly seen EOs, some novel EOs have also shown pesticidal properties. The EOs from *Cuminum cyminum* and *Pimpinella anisum* were toxic to the agricultural pests, *Myzus persicae* and *Spodoptera littoralis*, to the common housefly *Musca domestica* and to the lymphatic filariasis and Zika virus vector *Culex quinquefasciatus*, without affecting non-target invertebrates (189). The EO of *Eugenia uniflora* is toxic to the bronze bug, *Thaumastocoris peregrinus*, and selective to its parasitoid, *Cleruchoides noackae* (190).

Even though there have been thousands of studies conducted in which the bioactivities of EOs and their derivatives against insects were documented, most of those studies are limited to laboratory conditions and are on the R&D phase instead of the product development end. Therefore, only a handful of EO-based pesticides have been successfully commercialized, lagging far behind relative to the extensive scientific literature devoted to the area. Table 3 lists some of the current commercial EO-based insecticides/miticides.

**Table 3:** Currently available commercially formulated insecticides/miticides based on plant essential oils.

**Product**	**Producer**	**Active ingredient(s)**	**USDA organic compliant**	**Crop(s)**	**Target pest(s)**
EcoTrol™	EcoIPM (USA)	10% rosemary oil	Yes, OMRI listed	Vegetables Cucurbits Small fruits & Berries Citrus, Pome & Stone fruits Nuts Herbs & Spices	Whiteflies, spider mites, aphids, pacific mites,
EcoTrol^®^ Plus	KeyPlex (USA)	10% rosemary oil 5% geraniol 2% peppermint oil	Yes, OMRI listed	Vegetables Cucurbits Alfalfa Herbs & Spices Small fruits & Berries Grape & Hops Stone fruits & Pome fruits Citrus & Subtropicals Nuts Field crops Christmas trees Mushrooms	Aphids, beetles, early stages of caterpillars, flies, leafhoppers, leafminers, mealybugs, mites, softscales, thrips, whiteflies
GC-Mite™	JH Biotech (USA)	40% cottonseed oil 20% clove oil 10% garlic oil	Yes, OMRI listed	Berries Fruit trees Grapes Vegetables Melons & Cucurbits Peppermint & Herbs Flowers & Ornamentals	Mites (most spider mites, two-spotted mite, european red mite, Texas six-spotted spider mite, pacific mite, willamette mite, persea mite, rust mite, silver mite), thrips (avocado thrips, citrus thrips, flower thrips, greenhouse thrips), aphids (cabbage aphid, green peach aphid, black aphid, brown aphid)
Thyme Guard^®^	Agro Research International (USA)	23% thyme oil extract	Yes, Washington State Dept of Agriculture	All crops, turf, and ornamentals.	Sucking insects, such as psyllid, spider mite, scale and whitefly
Cedar gard™	Natural Resources Group (USA)	16% cedar oil	Yes, OMRI listed	Field crops Horticulture & Vegetables Grapes, tree fruit, citrus, nuts, berries, stone fruits Grass & Turf	Biting, sucking, and rasping insect pests
TetraCURB™	Kemin crop technologies (USA)	50% rosemary oil	Yes, OMRI listed	Food and non-food crops, indoor and outdoor production systems	Mites (such as spider mites), small, soft-bodied insects (such as aphids and whiteflies)
Trilogy^®^	Certis biologicals (USA)	70% clarified hydrophobic extract of neem oil	Yes, OMRI listed	Legume & Vegetables Nuts Small fruits & Berries Miscellaneous crops	Aphids, mealybugs, mites, soft scales, whiteflies, thrips
Eco-oil^®^	Organic Crop Protectants (AUS)	2% blend of tea tree (*Melaleuca*) and eucalyptus oils	Yes, ACO Certified (Australian Certified Organic Input)	Vegetables Ornamental plants Citrus Olive trees	Scale, aphids, two-spotted mite, whitefly, mealybugs and citrus leafminer
Akabrown^®^	Green Corp Biorganiks (MEX)	1.25% cinnamon oil 1.0% peppermint oil 0.5% clove oil 0.25% oregano oil	Yes, OMRI listed	Vegetables Berries	Spider mites

Isman (171) pointed out that commercial development of EO-based bioinsecticides can follow several different pathways, producing products with active ingredients including (1) a mixture of EOs; (2) a single EO, or a single terpenoid constituent; (3) a blend of synthetically produced terpenoids emulating a plant EO; and (4) non-natural blends of terpenoids obtained from different plant sources. Meanwhile, there are notable challenges for the commercialization of EO-based pesticides: (1) stability of EOs in storage and transport and persistence in field application; (2) residual action and efficacy after application; (3) phytotoxicity on crop and ornamental plants (191).

Formulation can be a potential method to improve EO-based pesticide performance. Formulation technologies, which involve using appropriate volumes of solvents and emulsifiers to make aqueous emulsions, can enhance the stability of non-polar EO constituents, thus increasing their efficacy and persistence, slowing down release, and preventing rapid evaporation. Nanoformulation technologies, including nano-emulsification and nanoencapsulation, are expected to improve EO chemical activity and persistence, and enable penetration into insect tissues, thus enhancing their insecticidal activity (192–194). In addition, almost any oil can be phytotoxic if applied as an aqueous emulsion at concentrations exceeding 2%, and in some cases, the phytotoxic concentration can be as low as 1%. Formulation is also a highly promising way to mitigate or eliminate phytotoxicity.

Three core constraints determining the potential for EO-based biopesticide production are (1) availability and consistency of the source plant, (2) cost, and (3) regulatory approval (171). The price largely depends on the availability of the EO materials. Widely used oils, such as for the fragrance and flavoring industries, produced on a massive scale can reduce the price level. In contrast, producing rarely used oils will result in extremely high prices for pest management products. One of the least expensive EOs is orange oil. The fruit peel used for extraction is a waste product of the orange juice industry, and the oil can be produced by cold pressing rather than hydrodistillation (191). Many EOs and some pure EO constituents are exempt from USA federal regulations on pesticides because of their minimal risk to human, animal, and environmental health (171, 191). While these low toxicity products are more likely to be allowed for use in organic systems, each product must be evaluated for compliance to the NOP federal rule prior to use in USA organic systems.

Overall, EO-based pesticides appear to be safer alternatives to conventional synthetic chemical insecticides in integrated pest management. They display toxic, repellent, and antifeedant activity against various insect and mite species. However, the commercialization of EO-based pesticides faces some challenges, including achieving necessary stability, persistence, efficacy, and mitigating phytotoxicity. Nanoformulation is a promising approach to improve the performance of EO-based pesticides. Further studies are required to develop novel formulations that would increase efficacy with lower costs.

### Weed Management

Weeds can interfere with the productivity of horticultural crops, reducing the yield and quality of fresh and processed products. Since the 1940s, when synthetic herbicides were discovered, their application has become the primary technique utilized for weed management (43). Conventional synthetic herbicides are water soluble, polar, and heat stable; therefore it is difficult to diminish and reduce the lethality (195). That causes evolution of herbicide-resistant weed biotypes, and soil and water pollution, which may eventually adversely influence humans. Thus, more selection of safe, efficient, and cost-effective organic herbicides, featuring diverse classes of compounds and having dissimilar mechanisms of action, are urgently needed.

The study of plant-plant interactions via the release of secondary metabolites could be a start for discovering of new substances with herbicidal activity (196). Essential oils and their constituents, primarily terpenoids, are good candidates as alternatives to synthetic herbicides. Utilization of EOs in weed management is mainly due to their allelochemical compounds (197). The term allelopathy refers to a plant-plant interaction, whereby allelochemicals released by one plant influence the physiological and biochemical processes of other surrounding plants directly or indirectly (198). Allelochemicals are a broad category of complex compounds that include, but are not limited to, EOs, carbohydrates, amino acids, salicylates, alkaloids, phenolics, flavonoids, jasmonates, momilactones, hydroxamic acids, brassinosteroids, and glucosinolates (199).

Generally, both monoterpenoids and sesquiterpenoids contribute to the phytotoxic and allelopathic effects of EOs (200, 201). However, their herbicidal activity often seems to be selective (202). For instance, at all doses tested, *Cistus ladanifer* EO totally inhibited *Amaranthus hybridus* germination and nearly stopped *Conyza canadensis* and *Parietaria judaica* germination. The EO had less impact on *Portulaca oleracea*, only limiting its germination at higher dosages tested. It had no effect on the germination of *Chenopodium album*. The EO displayed high phytotoxic activity in terms of seedling length, and it was effective at all concentrations tested (203). Whatever effect an active EO component has against one target species will not always be maintained against another species, even if they are in the same family or genus (202). This is a critical characteristic because herbicides require activity specificity. As a result, the primary task would be to discover the most effective chemicals for the various target weed species. Moreover, these compounds can function independently in certain situations, but also synergistically or antagonistically in others. Therefore, the nature of the interaction cannot be predicted in general based on the individual chemicals operating alone (202).

The phytotoxic and herbicidal potential of EOs against weeds has been extensively studied (Table 4). Essential oils have been investigated for their potential impact on seed germination rate and physiological growth. Rosemary EO, rich in 1,8-cineole, was found to significantly increase the amounts of proline and the relative membrane permeability of leaves, as well as strongly inhibit the germination of seeds and the growth of two weed species, amaranth weed (*Amaranthus retroflexus*) and radish (*Rhaphanus sativus*) (199). *Citrus aurantiifolia* EO and its major constituents, limonene (~41%) and citral (~28%), were demonstrated to affect mitotic activity and induce chromosomal abnormalities in three grassy agricultural weeds [*Avena fatua, Echinochloa crus-galli*, and *Phalaris minor*; (235)]. Citral was shown to be the most toxic followed by *C. aurantiifolia* oil and limonene through phytotoxicity and cytotoxicity assays. However, not all EOs display an herbicidal effect. For example, *Achillea gypsicola* EO was shown to be ineffective against the germination of *Chenopodium album* and *Rumex crispus* seeds (236). Moreover, EO from *Pinus radiata* was found to exhibit an herbicidal effect that was significantly more effective on dicots than monocots (222). The herbicidal potential of *Thymbra capitata, Mentha* × *piperita*, and *Santolina chamaecyparissus* EOs were evaluated on *Avena fatua, Echinochloa crus-galli, Portulaca oleracea*, and *Amaranthus retroflexus*. Both *Thymbra capitata* and *Mentha* × *piperita* EOs showed a broad spectrum of activity, with *Thymbra capitata* at the highest doses applied (12 μL mL^−1^) killing plants of all weed species except for *Portulaca oleracea* at 90%. *Mentha* × *piperita* at the highest dose (20 μL mL^−1^) completely controlled *Avena fatua* and *Amaranthus retroflexus* plants but displayed 90 and 40% efficacy on *Portulaca oleracea* and *Echinochloa crus-galli*, respectively. Although *Santolina chamaecyparissus* EO was less active than the other EOs, it demonstrated an excellent selective activity, being highly effective against *A. retroflexus*, showing 90% efficacy at the highest dose, 20 μL mL^−1^ (230).

**Table 4:** Examples of EOs presenting herbicidal properties.

**Essential oil distilled from**	**Tested plant**	**References**
*Ambrosia artemisiifolia*	*Poa annua*	(204)
	*Setaria viridis*	
	*Amaranthus retroflexus*	
	*Medicago sativa*	
*Artemisia fragrans*	*Convolvulus arvensis*	(205)
*Baccharis* spp.	*Lactuca sativa*	(206)
	*Bidens pilosa*	
*Carum carvi*	*Echinochloa crus-galli*	(207)
*Chromolaena odorata*	*Echinochloa crus-galli*	(208)
	*Amaranthus viridis*	
*Copaifera especies*	*Mimosa pudica*	(209)
*Eryngium triquetrum*	*Lepidium sativum*	(210)
*Smyrnium olusatrum*		
*Eucalyptus citriodora*	*Angallis arvensis*	(211)
*Ocimum basilicum*	*Cyperus rotundus*	
*Mentha arvensis*	*Cynodon dactylon*	
*Eucalyptus globulus*	*Portulaca oleracea*	(212)
*Foeniculum vulgare*	*Triticum aestivum*	(213)
*Lavandula angustifolia*	*Lolium multiflorum*	(214)
*Litsea pungens*	*Lolium perenne*	(215)
	*Bidens pilosa*	
*Mentha longifolia*	*Cyperus rotundus*	(216)
	*Echinochloa crus-galli*	
*Monarda didyma*	*Papaver rhoeas*	(217)
	*Taraxacum officinale*	
	*Avena fatua*	
	*Raphanus sativus*	
	*Lepidium sativum*	
*Nepeta flavida*	*Lepidium sativum*	(218)
	*Raphanus sativus*	
	*Eruca sativa*	
*Ocimum basilicum*	*Abutilon theophrasti*	(219)
*Thymus vulgaris*		
*Melissa officinalis*		
*Origanum vulgare*	*Amaranthus retroflexus*	(220)
*Rosmarimum officinalis*	*Portulaca oleracea*	
	*Convolvulus arvensis*	
	*Eruca sativa*	
	*Papaver rhoeas*	
*Pimpinella anisum*	*Anagalis arvensis*	(221)
	*Malva parviflora*	
*Pinus radiate*	*Sinapis arvensis*	(222)
	*Trifolium campestre*	
*Piper cubeba*	*Bidens pilosa*	(223)
*Piper nigrum*	*Echinochloa crus-galli*	
*Pogostemon benghalensis*	*Avena fatua*	(224)
	*Phalaris minor*	
*Ruta graveolens*	*Amaranthus retroflexus*	(225)
*Citrus bergamia*	*Convolvulus arvensis*	
	*Rumex crispus*	
*Salvia rosmarinus*	*Acacia saligna*	(226)
*Satureja hortensis*	*Amaranthus retroflexus*	(227)
	*Chenopodium album*	
*Tagetes erecta*	*Echinochloa cruss-galli*	(228)
*Thymbra capitata*	*Erigeron canadensis*	(229)
	*Sonchus oleraceus*	
	*Chenopodium album*	
	*Setaria verticillata*	
	*Avena fatua*	
	*Solanum nigrum*	
	*Amaranthus retroflexus*	
	*Portulaca oleracea*	
	*Echinochloa crus-galli*	
*Thymbra capitata*	*Avena fatua*	(230)
*Mentha × piperita*	*Echinochloa crus-galli*	
	*Portulaca oleracea*	
	*Amaranthus retroflexus*	
*Thymus eigii*	*Lactuca sativa*	(231)
	*Lepidium sativum*	
	*Portulaca oleracea*	
*Thymus kotschyanus*	*maranthus retroflexus*	(232)
	*Panicum miliaceum*	
*Thymus proximus*	*Amaranthus retroflexus*	(233)
	*Poa anuua*	
*Trachystemon orientalis*	*Cuscuta campestris*	(234)

*Only treatments that displayed statistically significant herbicidal effects are included; consult the references for treatment details*.

Regarding phytotoxic effects of EO, visible symptoms such as inhibition of germination and seedling development, along with necrosis and chlorosis (237), or leaf burning (238), were previously reported to be related to the following mechanisms of action (43, 239).

(1) Essential oils and their pure components induce oxidative damage and loss of membrane integrity by generation of ROS, causing ion or electrolyte leakage, membrane depolarization, cuticular wax interruption, stomata clogging, and epidermal cell shrinkage. Citronellol was reported to inhibit root and shoot growth of *Triticum aestivum* by ROS-mediated membrane disruption. ROS production could result in lipid peroxidation, membrane damage, and solute leakage (240). As a consequence of the disruption of the membrane integrity, Citronellal-treated weed leaves showed cuticular wax interruption, stomata clogging, epidermal cell shrinkage, and rapid electrolyte leakage (241).

(2) Inhibition of DNA synthesis and mitosis. It is widely believed that EO phytotoxic effects are mediated by suppression of DNA synthesis, interfering with mitotic activity, or disrupting surrounding membranes of mitochondria and nuclei in some organelles such as mitochondria (242). The compound 1,8-cineole, the major component of rosemary and mentha EOs, was suggested to inhibit DNA synthesis and mitotic activity in both cell nuclei and organelles in root apical meristem of *Brassica campestris* (243, 244).

(3) Reduction of cellular and mitochondrial respiration. Most of the fundamental cellular activities, including cell division, ion and solute transportation across membranes, and synthesis of molecules such as membrane lipids, chlorophyll, proteins, and nucleic acids, require a source of metabolic energy. Mitochondrial respiration supplies ATP to support these processes (239). Abrahim et al. (245) confirmed that α-pinene, a major component of rosemary EO, strongly impaired mitochondrial energy metabolism and inhibited ATP production of maize seedlings by uncoupling of oxidative phosphorylation and inhibition of electron transfer. They further illustrated that α-pinene can inhibit or completely suppress mitochondrial respiration depending on its dose.

(4) Inhibition of photosynthesis. It was demonstrated that the EOs from *Cymbopogon nardus* and *Eucalyptus citriodora* as well as pure citronellal reduced chlorophyll content and total protein content in crabgrass (*Digitaria horizontalis*) and burrgrass (*Cenchrus echinatus*) by more than 80 and 90%, respectively (201). *Origanum vulgare* EO was confirmed to negatively affect nitrogen assimilation into glutamine, causing excessive accumulation of toxic ammonia in leaf cells as well as oxidative stress. Afterward, a series of events that suppress the effectiveness or efficiency of PSII, producing oxidative stress and, ultimately, a significant decrease in plant growth and development, followed by leaf necrosis and plant death (246).

(5) Microtubule polymerization. Chaimovitsh et al. (247) indicated that both limonene and citral could disrupt microtubules and that limonene could also cause membrane leakage. Limonene displayed dual capacity in terms of microtubules and membrane functionality.

(6) Proline accumulation and lipid peroxidation. In plants, proline functions as a mediator of osmotic adjustment and plasma membrane integrity and protection. Its accumulation could be related to the increase of protein hydrolysis caused by stress (248). The phytotoxicity of various EOs on weeds can be determined by the expression and accumulation of oxidative stress products, such as proline and lipid peroxidase. Khare et al. (211) found mentha EO emulsion injures weeds by disturbing the membrane integrity and inducing oxidative stress to the weed as indicated by relatively higher levels of electrolyte leakage, proline and lipid peroxidase. In general, EOs inhibit electron flow in mitochondria, resulting in an increase in the generation of ROS, which promotes lipid peroxidation. It is possible that membrane breakdown might result in lipid release inside the cytoplasm of targeted cells, because fatty acids and other lipids are known to be structural components of membranes. The liberated lipids in the cytoplasm might then become the target of oxidative activity (211). *Cymbopogon citratus* EO also demonstrated strong phytotoxic activity against barnyard grass (*Echinochloa crus-galli*) by increasing relative electrolyte leakage and lipid peroxidase activity (249).

Essential oils are promising candidates for the development of novel bio-herbicides due to their strong phytotoxic activity. Currently available EO-based commercial organic herbicides are listed in Table 5. The primary challenges are (1) developing appropriate formulations to minimize their high volatility and optimize effectiveness while permitting field application. So far, most of the tests are done in a small scale under laboratory conditions. (2) determining their modes of action. Because EOs are a complex mixture of biologically active molecules capable of affecting multiple targets at the same time, they might be useful in preventing the formation of resistant weeds. Combining the classical analytical techniques with new -omics approaches, such as genomics, transcriptomics, proteomics, and metabolomics, would speed up discovering new mechanisms of action (196).

**Table 5:** Currently available commercially formulated organic herbicides based on essential oils or plant extracts (196, 250).

**Product**	**Source**	**Main component**
GreenMatch	Marrone Bio Innovations (CA)	55% d-limonene
Matratec	Brandt Consolidated (IL)	50% clove oil
WeedZap	JH Biotech (CA)	45% clove oil + 45% cinnamon oil
GreenMatch EX	Marrone Bio Innovations (CA)	50% lemongrass oil
Avenger Weed Killer	Avenger Products (GA)	70% d-limonene
Weed Slayer	Agresearch International (TX)	6% eugenol
BioWeed	Barmac (AUS)	derived from *Pinus radiata*
Beloukha	Grochem (AUS)	derived from *Brassica napus*

In the future, a better understanding of mechanisms of action would promote the development of bio-herbicides, especially focusing on the potential synergism among single molecules. Therefore, it may help to reduce the herbicides application doses, avoid herbicide resistance in weeds, and hit multiple targets simultaneously.

## Essential Oil Formulations and Applications in the Food Industry

Despite proven efficacy of EOs, they still do not enjoy widespread application due to their high volatility, low stability, low water solubility, composition variability, a strong influence on organoleptic properties, and phytotoxic effects. Essential oils are also very sensitive to light radiation, especially UV, and elevated temperature, which could cause oxidation, isomerization, polymerization, dehydrogenation, and eventually degradation (251). The degradation may alter the biological properties of the EO as well as exerting potent toxicity due to the presence of alteration compounds (43). Due to these limitations, many EOs are not suitable for use in their raw form (252). To break through these limitations and improve the efficiency and persistence of EOs, formulation techniques applied to EOs has a bright and promising future. A product formulation is a homogeneous and stable combination of an active ingredient and inactive materials as additives that involve specialized processing of the product to improve its biological qualities, durability, and stability (253). Formulations are commonly used for pesticides and herbicides. A recent study demonstrated that polydopamine microcapsules templated by Pickering emulsions stabilized by cinnamoyl chloride modified cellulose nanocrystals for EO encapsulation. The EO (turpentine) functioned as a botanical pesticide and a solvent for the herbicide. The system improved active ingredients encapsulation efficiency, exhibited excellent multi-active ingredients encapsulation, adhesive and UV resistance properties, and controlled release of active compound (254). Therefore, formulation technique should be applicable to EOs in the food industry to achieve comparable advantages (43). In this section, non-compliant and compliant raw materials, processing methods, and formulations (products) are discussed to allow for a more complete presentation of essential oils and their applications.

### Emulsification

An emulsion is defined as a combination of two immiscible liquids, containing spherical droplets as the dispersed phase and the addition of surfactant as the continuous phase (255). Emulsions are primarily classified based on their particle size and kinetic stability as macroemulsions (or coarse emulsions), microemulsions, and nanoemulsions (256). Macroemulsions are opaque and are thermodynamically metastable and susceptible to breakdown. Microemulsions are transparent and thermodynamically stable, but their stability is affected by slight environmental condition variations such as composition and temperatures. Nanoemulsions are thermodynamically metastable as phase separation occurs over time, however, they have kinetic stability because there is no gravitational separation and droplet aggregation due to the attractive force between the tiny sized droplets is minimal (255, 257).

Emulsion-based delivery systems can be formulated with food-grade ingredients to disseminate EOs to areas where microorganisms grow and proliferate (258). The nanoemulsions are more suitable than microemulsions or free EOs for these applications because their kinetic stability is not affected by physical and chemical variations, such as temperature and pH. Thus, they need fewer surfactants for preparation and are more cost-effective (255, 259). Furthermore, because of their subcellular size and improved diffusion, nanoemulsions can improve product physicochemical stability and reduce the influence on the organoleptic characteristics of foods while increasing bioactivity (259).

Edible coatings and films based on nanoemulsions of EOs have been investigated to prolong fresh produce shelf life. Several main types of matrices were usually used as the base to create EO nanoemulsions for use as edible coatings. These matrices have included starch, chitosan, sodium alginate, hydroxypropyl methylcellulose, and carnauba wax (260). As EOs are emulsified, they have been found to have higher stability and lower influence on food sensory qualities, and reduced interaction with other food matrix ingredients, while delivering enhanced biological activity due to the increased surface area and small droplet size (261). It was also demonstrated that for a film-forming formulation the addition of lemongrass EO emulsions by emulsification with two emulsifiers (Tween 80/pectin) to glycerol-plasticized cassava starch resulted in both EO and emulsifier phases showing good interaction and compatibility. Emulsification caused crucial changes in the active starch films, such as improved colorimetric attributes, thermal stability, moisture barrier properties, and mechanical properties (increasing extensibility, resistance, and stiffness). This film-forming formulation further increases the potential application of EO in the food packaging industry (262).

To summarize, nanoemulsion-based delivery systems could be a suitable method for developing EO products with higher kinetic stability and bioactivity, less organoleptic impact, and more cost-effectiveness than free EOs.

### Encapsulation

Essential oils include components that are extremely sensitive to volatilization, and chemical changes due to oxygen, light, humidity, and heat exposure (263). Hence, EO encapsulation has been widely utilized to protect those sensitive compounds from undesired conditions, and improve their activity duration and functional performance (264). Moreover, encapsulation increases EO solubility, and provides targeted delivery and controlled release of EOs (265). The process of enveloping a particle or molecule of interest with a coating or constructing a functional barrier between a bioactive core and wall material in order to minimize physical and chemical interactions between the core and the outside molecules is referred to as EO encapsulation (266). Generally, polymeric particles (50), liposomes (267), and solid lipid nanoparticles (268) have been used as wall materials to protect the EOs from degradation.

Polymeric particles such as methacrylate polymer (269), poly(D,L-lactic-co-glycolic acid) (PLGA) (270), poly-ε-caprolactone (PCL) (271), poly (lactic acid) (PLA) (272), poly (L-lactide-co-ε-caprolactone)/silk fibroin (PLCL/SF) (273), β-cyclodextrin (274), cellulose nanofibrils (275), alginate (276), starch (277), chitosan (278), gum arabic, maltodextrin, and inulin (279), were commonly used as wall materials individually or as a mixture. For EO encapsulation in polymeric particles, spray drying, coacervation, nanoprecipitation, and rapid expansion of supercritical solutions (RESS) are widely used technologies (263).

Spray drying is a popular method of producing microparticles since it is a simple, rapid, and reproducible technique that produces stable final products with lower cost and allows continuous industry-scale production (280–282). In this process, the mixture solution of the active ingredient and the encapsulating materials are fed to the spray-dryer and atomized by hot gas, resulting in extremely rapid water evaporation and, as a result, quasi-instantaneous entrapment of the EO in a rapid-formed crust (43, 283).

Coacervation is defined as the phase separation of one or many hydrocolloids from the initial colloidal solution (284). One phase is rich in polymer and is known as the coacervate phase, while the other does not contain polymer and is known as the equilibrium solution. Coacervation techniques can be simple or complex depending on the number of polymers used. There is just one polymer in simple coacervation, but complex coacervation involves the interaction of two oppositely charged colloids (263).

Nanoprecipitation, also known as solvent displacement or interfacial deposition, was invented by Fessi et al. (285). Two miscible phases are involved: an organic phase (the solvent) and an aqueous phase (the non-solvent). This method is suitable for encapsulating hydrophobic compounds such as EOs. For EO encapsulation by nanoprecipitation, the polymer and the EO are solubilized in an organic solvent, then added to water under moderate magnetic stirring, which causes the interfacial deposition of a polymer after the organic solvent has been displaced. Afterward, the organic solvent is evaporated with a rotavapor to form the nanoparticles suspension in water (286).

The RESS procedure has recently been regarded as an effective method for producing free-solvent particles with consistent morphology and size distribution (287). Supercritical carbon dioxide (ScCO_2_) is considered to be a promising supercritical fluid for use in RESS because it is non-toxic, inflammable, and cheap, with a low critical temperature (31.3 °C) and pressure (73.8 bar) (288).

Liposomes are colloidal, vesicular structures composed of one or more phospholipid bilayers that define one or more aqueous compartments surrounded by a lipid membrane. Phospholipids are amphiphilic molecules that spontaneously self-assemble in aqueous environments (263). These liposomes form sphere-like shells and encapsulate hydrophilic compounds (such as EOs) as well as lipophilic or even amphiphilic molecules in the inner aqueous phase with oil-soluble substances in the lipid bilayer membrane (289). Encapsulation of EOs in liposomes often uses thin film hydration, reverse phase evaporation, and supercritical fluid methods (263). The thin film method, first developed by Bangham et al. (290), is one of the most widely used and simplest techniques for formulating of liposomes. However, due to low production capacity, the presence of organic solvent residues in the final product, and heterogeneous size distribution, this technique has limited commercial applicability (291). Several approaches are used to homogenize and reduce size of liposomes formed by the thin film hydration method, such as sonication, extrusion, and freeze-thaw (263). The extrusion refers to multiple times passage of heterogeneous sized particles via a track-etched polycarbonate membrane with holes of varying sizes, including hot-melt extrusion, melt injection extrusion process, co-extrusion, and electrostatic extrusion (292). Freeze-thaw cycles increase interactions between the lipid film and the EO to incorporate and display high encapsulation efficiency (293). The process of reverse phase evaporation involves mixing a phospholipid organic phase with the lipophilic active substances in an aqueous phase to form oil-in-water emulsion. Then the organic solvent is evaporated, yielding large unilamellar vesicles (294).

Two methods for supercritical fluid technology are modified RESS and particles from gas saturated solutions (PGSS)-drying of emulsion (263). In the process of modified RESS, liposomal materials and EO are dissolved in a supercritical CO_2_/ethanol solvent, and the solution is then sprayed into a buffer solution using a coaxial nozzle to create a liposome suspension (295). The process of PGSS was to create encapsulated particles by saturating the suspension of the bioactive chemical and the wall material with CO_2_ at a proper pressure and temperature to lower the viscosity. At atmospheric pressure, the vaporization and expansion of CO_2_ are triggered through a nozzle, which creates an intense cooling effect, resulting in forming a very fine and dry powder (296).

Solid lipid nanoparticles (SLNs) are nanocarriers containing lipids or lipid-like molecules, solid at room temperature. Compared to other colloidal carriers, liquid lipid is replaced by solid lipid in SLNs. The use of solid lipid rather than liquid lipid is advantageous since it has been demonstrated to enhance control over the release kinetics of encapsulated chemicals and the stability of integrated chemically-sensitive lipophilic components (297). Solid lipid nanoparticles can improve the stability and solubility of EOs in water, avoid the use of organic solvents, display high drug payload and no biotoxicity of the carrier, increase the bioavailability of entrapped bioactives, provide controlled release of EOs, and favor large scale production. However, SLNs also demonstrate some disadvantages, such as high pressure-induced drug degradation, lipid crystallization and drug incorporation, and unpredictable gelation phenomena (298). Solid lipid nanoparticles are mainly prepared by high pressure homogenization or micro emulsification (297).

For the preservation of fruits and vegetables, encapsulation methods have been extensively researched. Protein/polysaccharides were usually used as wall materials of EO microcapsules, such as β-cyclodextrin, pectin, chitosan, starch–gellan, alginate, sodium alginate, gelatin, and carboxymethyl cellulose (299). In a recent study, lemongrass EO-containing poly(lactic acid) nanocapsules were made to achieve long-term thermal stability of the EO. Compared to the apples treated with non-encapsulated lemongrass EO, the fruit treated with encapsulated EO showed three times smaller bitter rot lesions (272). López-Gómez et al. (300) tested the effect of different sized active packages (including β-cyclodextrin-EOs inclusion complex) on the quality of grapes, nectarines, and lettuces, representing berry fruit, stone fruit, and leafy vegetables. The EOs–β-cyclodextrin inclusion complex was dissolved in water-diluted lacquer, then sprayed on all internal surfaces of the packages. They found active cardboard packages with greater active surface better preserved quality of grapes, nectarines and lettuce, for which the sensory quality was acceptable after 30, 25, and 14 days, respectively.

To sum up, encapsulation could be an effective formulation method, which could address some of the drawbacks associated with the usage of EOs in their raw form. In particular, encapsulation protects EOs from light, air, and humidity, allows targeted delivery and controlled release of EOs, boosts EO solubility and bioactivities, and reduces degradation during storage, such as through oxidation or volatilization.

### Edible Coatings

Edible coatings have attracted great attention among various postharvest packaging technologies due to their cost-effectiveness, environmentally friendly characteristics, and ability to carry bioactive substances. An edible coating is defined as a thin layer of edible material on a food surface, which can be digested by the human body or are biodegradable in the environment, used as a barrier to protect the product from physical damage and chemical reactions (301, 302). So far, edible coatings are widely used on whole or fresh-cut fruits and vegetables to prolong their shelf life and maintain their sensory qualities.

Different kinds of EOs, such as ginger (303), oregano (304), cinnamon (305), lemongrass (306), and lavender (307) have been incorporated into edible coating as anti-microbial agents. Direct application of EOs to fresh commodity surfaces is limited due to their instability, hydrophobicity, and organoleptic effect. However, emulsification and encapsulation of EOs that is compatible with coating systems can maintain the bioactivity, control the release, increase the effective rate, minimize the organoleptic impact, and increase the contact area and duration on food surfaces (308).

The name, EO-edible coatings, refers to coatings in which a thin layer of a mixture of EOs and biological polymers that are able to carry EOs are used to create a food coating. Three types of biological polymers are commonly used for EO-edible coatings: polysaccharides, proteins, and lipids (301). Natural gum, chitosan, starch, pectin, and alginate are commonly used polysaccharides for EO-edible coatings. The protein-based coatings typically contain gluten, collagen, zein, casein, or whey proteins. The lipid-based coatings mainly include wax, acyl glycerol, and fatty acids (301). In addition, composite coatings refer to the combination of water colloids and lipids, which could provide multiple benefits (309, 310). Four manufacturing methods of EO-edible coatings are: (1) dipping (for thick and uniform materials), (2) spraying (for thin and uniform materials), (3) spreading (for medium thickness and a little uniform materials), and (4) thin film hydration (for poor uniformity of liposome particle size materials) (301).

In brief, EOs encapsulated in edible coating have better stability and show good fresh-keeping potential. Further research should keep exploring novel combinations of basic materials and EOs for coatings that can be used on a variety of food items, which are safe, economic, and effective.

## Future Prospects

Overall, EOs demonstrate antimicrobial, pesticidal, and herbicidal effects in both *in vitro* and *in vivo* studies. They display great potential in organic product cultivation and food preservation. However, most of the studies that have been conducted are limited to laboratory conditions and in-field analysis of product efficacy is needed. Currently, the number of commercially available biocontrol products using EOs as active components is limited. The reasons could be (1) Their effectiveness is selective. Their efficacies are variable among different pathogen species, sometimes even within the same pathogen species but different strains. (2) EOs can be a double-edged sword. They may display anti-fungal effects and phytotoxicity effects at the same time. It would be hard to find a proper application concentration for every crop at every developmental stage that can totally suppress the pathogens without damaging the plants. 3 Our understanding of the anti-pathogen, pesticidal, and herbicidal effects of EOs and their components is still developing. More work is needed to elucidate their modes of actions, cost-effectiveness, and potential impacts on non-targeted species. There is enormous potential for selecting plants to produce EOs with enhanced efficacy, such as blending EOs to achieve synergistic effects or a broader spectrum of action and formulated with other materials to extend residual effects. As people are more concerned about human and environmental health and are willing to pay more for organic commodities, theoretical and practical studies of EOs should continue simultaneously and more focus should be placed on the development, extension, and market entry of newly formulated EO products.

In the food industry, EOs have great potential to be used as organic food preservatives. Nanoemulsions of EOs have been found to enhance quality and shelf life of fresh commodities. Encapsulation should be used to mask the strong organoleptic impact of EOs while maintaining their preservative effects. Nevertheless, the pungent odor of EOs will remain a big problem. Some valuable research topics can include methods to minimize the odor of EOs or discover effective EO combinations in which the unique EO odor is compatible with or enhances the original food flavor. Furthermore, since EOs are relatively expensive, the development of economic, simple, and stable EO extraction techniques on an industrial scale would support product innovation and expand adoption.

## Author Contributions

YC, JB, and AS conceived and designed the structure of the manuscript. YC conducted the literature review and primarily wrote the manuscript. JB, AS, DC, PH, and DT reviewed and edited the manuscript and contributed additional literature and writing. JB and AS revised the manuscript. All authors contributed to the article and approved the submitted version.

## Funding

This work was supported by award 2020-51300-32181 from the USDA National Institute of Food and Agriculture, Organic Agriculture Research and Extension Initiative program.

## Conflict of Interest

The authors declare that the research was conducted in the absence of any commercial or financial relationships that could be construed as a potential conflict of interest.

## Publisher's Note

All claims expressed in this article are solely those of the authors and do not necessarily represent those of their affiliated organizations, or those of the publisher, the editors and the reviewers. Any product that may be evaluated in this article, or claim that may be made by its manufacturer, is not guaranteed or endorsed by the publisher.
